# Permanent Deformation and Rutting Resistance of Demolition Waste Triple Blends in Unbound Pavement Applications

**DOI:** 10.3390/ma14040798

**Published:** 2021-02-08

**Authors:** Farshid Maghool, Muditha Senanayake, Arul Arulrajah, Suksun Horpibulsuk

**Affiliations:** 1 Department of Civil and Construction Engineering, Swinburne University of Technology, Melbourne 3122, Australia; ssenanayake@swin.edu.au (M.S.); aarulrajah@swin.edu.au (A.A.); 2 School of Civil Engineering, and Center of Excellence in Innovation for Sustainable Infrastructure Development, Suranaree University of Technology, Nakhon Ratchasima 30000, Thailand and Academy of Science, The Royal Society of Thailand, Bangkok 10300, Thailand

**Keywords:** pavement base, permanent deformation, wheel-tracker, rutting, demolition wastes, ground improvement

## Abstract

Virgin quarried materials are becoming increasingly scarce in our environment, and these materials are no longer a viable or economical solution for the construction industry. The construction industry is constantly seeking new markets for recycled waste in civil engineering applications. This research’s primary focus is the optimization of the usage of recycled materials such as recycled glass (RG), crushed brick (CB), and crushed concrete (CC), in pavement base/sub-base applications. Various percentages of RG, up to 40%, were blended with two types of CC in this research. The CC and CB, which were used as triple blends in this research, were utilized for the upper (100% CC) and lower sub-bases (up to 50% CB). This study sought to establish the maximum amount of RG that could be incorporated in the triple blends with CB and CC whilst maintaining an acceptable performance. Thus, a comprehensive series of fundamental and advanced geotechnical laboratory tests, including repeated load triaxial (RLT) and wheel-tracker (WT) tests, were performed to assess the engineering properties and permanent deformation characteristics of these triple blends. The particle-size distribution curve and California Bearing Ratio (CBR) values of all the blends met the minimum requirements. Results of RLT tests confirmed that all the nominated blends were found to provide the resilient modulus value required to be used as pavement materials. The WT results on the triple blend with 15% RG showed that the specimen performed exceptionally well during the test and comfortably met the requirements to be used in pavement applications. Based on the engineering properties and permanent deformation results, up to 15% RG can be suggested for incorporation as an accompanying material in unbound roadwork applications. Subject to the outcomes of future field testing, there might be potential to increase the percentage of RG added in the blends up to 30%.

## 1. Introduction

The use of natural resources in major construction projects is not only costly, but increasingly unsustainable. Natural resources are becoming very limited in supply, which is detrimental to the environment. In recent years, researchers have established that recycled materials can offer improved performance, and in some cases, more durability compared to virgin quarried materials [1]. This direction, using recycled materials instead of virgin materials, will inevitably reduce landfills and carbon footprint. Effectively employing recycled materials is a viable sustainable solution, ultimately retaining natural resources [2].

In recent years, the disposal of solid waste has become a major problem, and has become increasingly expensive. Waste levies are imposed and intended by state governments in Australia to support moving waste materials from landfill to recycling [3]. For instance, levies for disposal at landfill in Australia’s major cities for municipal solid waste are around AUD$90 per ton, for construction and industrial waste they are AUD$146 per ton, and levies for mixed waste are AUD$199 per ton [3]. Even recycling plants are accumulating large stockpiles of recycled materials.

The designers and relevant authorities for infrastructure projects globally have, until now, favored working with naturally occurring materials. Before they can accept any new materials, the performance and service life of these recycled materials need to be carefully tested under simulated traffic loading, and different environmental and climatic conditions. The determination of critical parameters such as the resilient modulus and permanent deformation, using the repeated load triaxial (RLT) test is of increasing interest to design engineers and decision makers. Rutting resistance is one of the main concerns in pavement design. Rutting can occur due to the continued growth of permanent deformation during the lifetime and serviceability of the pavement structure [4]. Meeting the requirements for good resistance rutting in granular layers is more difficult than developing an acceptable resilient modulus [5]. The wheel-tracker (WT) test can provide an accurate estimation of rutting for designers and simulate real traffic loading on pavement.

To date, several attempts have been made to substitute virgin materials with recycled materials such as construction and demolition materials including recycled glass (RG) [6,7], crushed concrete (CC) [8,9], crushed brick (CB) [10,11], crushed waste rock [12,13], recycled plastics [14,15], reclaimed asphalt pavement [16,17], biosolids [18], coffee [19], crumb rubber [20,21], light-weight foamed glass [22], and different types of slags [23,24,25]. Lately, significant volumes of these materials have been well-utilized as new construction materials in different applications, such as typical civil, pipe bedding, rural road, and fill applications.

Some studies have shown that up to 20–25% RG can be added to natural unbound material without substantial effects on the mechanical behavior of base course layers [26]. Some research proved that up to 25% CB could be securely added to CC and crushed-rock blends in pavement unbound sub-base applications [27]. Some studies permitted up to 50% CB content for sub-base applications after treating with 3% cement [28].

In the state of Victoria alone, approximately 300,000 tons of RG, 1.4 million tons of CB, and 3.5 million tons of CC are available for processing per annum. RG, CC, and CB are well-known recycled materials in the construction industry. These materials have been shown to exhibit promising results in regard to engineering properties and have the potential to substitute natural quarry in a number of road applications [29,30]. Assessments of the environmental risks of RG, CC, and CB have also been conducted and compared to environmental protection policies. These materials were determined not to exhibit any leaching hazards during their service life in roadwork applications [29,30]. This study proposes to investigate the use of RG (up to 40%) with two types of CC in upper (100% CC) and lower sub-bases (comprising up to 50% CB) as triple blends in unbound roadwork applications. This study seeks to establish a new standard figure for the maximum amount of crushed glass that can be incorporated in triple blends whilst maintaining acceptable performance. The permanent deformation characteristics of the triple blends were determined and compared using RLT and WT tests.

## 2. Materials and Methods

**Figure 1:**
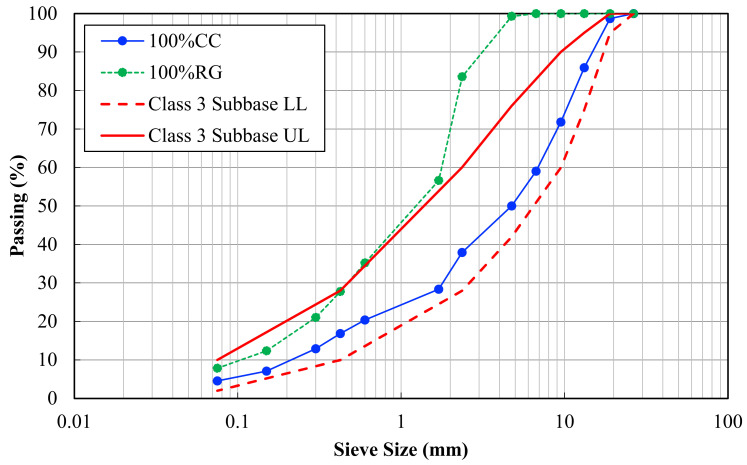
Particle size distribution of the parent materials.

Different percentages of RG, up to 40%, were triple-blended with CB and CC in this research. The CC utilized was for the upper (100% CC) and lower sub-bases, which in the latter case was comprised of up to 50% CB. All recycled materials for this study were obtained from a major local recycling company in Melbourne, Australia. Particle size distribution testing was conducted following AS 1141.11 “Particle size distribution by sieving” [31]. Sieves were individually weighed before testing to ensure minimal material loss during the testing phase. For the sieving process, a minimum material mass of 3 kg was utilized, and the resulting particle size distribution curve was plotted for each material. Improved grading provides maximum compacted density for granular material in order to maximize resistance to rutting [5]. Figure 1 illustrates the particle size distributions of the parent materials used in this research along with the upper limits (UL) and lower limits (LL) of the local road authority.

The particle density test was performed for both the fine-fraction AS 1141.5 [32] and coarse-fraction AS 1141.6.1 [33] of all recycled materials. The organic content of each material was determined using ASTM D2974 “Standard test methods for moisture, ash, and organic matter of peat and other organic soils” [34]. The flakiness index test was conducted to evaluate the overall thickness parameters in relation to the material sample size in accordance with AS 1141.15 [35]. Los Angeles (LA) abrasion tests were performed to evaluate the resistance of the parent materials to abrasion following ASTM C131 [36].

The optimum moisture content (OMC) and maximum dry density (MDD) of all the nominated blends being tested were evaluated using the modified compaction method, which was performed based on the AS 1289.5.2.1 [37]. The samples were compacted at varying moisture contents using five separate layers within a metal mold 105 mm in diameter and 115.5 mm in height. Once compaction was completed, samples were weighed pre- and post-drying to determine the moisture content. The dry density and moisture content data for the varying samples were then plotted and graphed in order to determine the OMC and MDD for each individual mixture.

The California Bearing Ratio (CBR) tests were performed to determine the soil strength capabilities of mixtures compacted at 98% MDD and 100% OMC, as determined in the “modified compaction” testing. Samples were compacted following AS 1289.6.1.1 “Soil strength and consolidation tests—Standard laboratory method for a remolded specimen” [38]. All nominated blends were compacted in molds 152 mm in diameter and 117 mm in height at 100% MDD and 98% OMC, as specified by the Australian Standards. Each mixture consisted of 5 separate (scratched) layers and were compacted individually with 56 weighted blows. Once compacted, samples were weighted and then submerged in a water bath for four days prior to testing.

The RLT test was performed to assess the deformation and resilient modulus (M_R_) characteristics of the selected mixtures, following the Austroads Test Method AG:PT/T053 [39]. During the test, samples are exposed to a series of loading cycles to recreate the pavement dynamic loads influenced by moving vehicles [40]. Each sample was compacted at 100% OMC and MDD within a metal mold 100 mm in diameter and 200 mm in height. The mixture compaction consisted of 25 individual blows for each of the 8 (scratched) layers. After compaction, the samples were dried back to 70% OMC prior to RLT testing. The permanent deformation tests were carried out at three loading stages with a constant confining pressure of 50 kPa and deviator stresses of 350, 450, and 550 kPa. Each loading cycle consisted of 1 s of rectangular loading followed by a 2-s resting period. A constant positive minimum deviator stress of 5 kPa was also maintained on top of the sample to exclude the possibility of sudden loading. Fully automated cyclic triaxial equipment with a maximum capacity of 16 kN was used. The axial deformation of the sample was measured at each load cycle by three axial linear variable differential transformers (LVDT) mounted on the loading rod. Confining pressure was applied with confined air and was controlled through an air-pressure transducer. A total of 7 tests were carried out to determine the effect of RG inclusion on the permanent deformation of blends.

WT tests are widely used to assess the rutting sensitivity of crushed material mixes. In WT tests, a slab of material is exposed to rolling passes of a loaded wheel for 1000 cycles. This causes material rutting under the wheel. The rut depth is measured as a function of the number of cycles. For a given number of cycles, the rut depth reached provides a relative assessment of material rutting resistance [41]. The WT test was executed via a real-sized wheel with an inflated tire pressure of 600 kPa, applying an axial load of 8 kN onto two samples in accordance with AGP-T054 [42]. The wheel was travelling at a frequency of 0.4 Hz and had a diameter of 550 mm and a width of 110 mm. The subsequent load applied by the WT test can therefore cause deformation within the samples, and this information was recorded by an automated laser scanning device. The laser recorded the information at the end of each loading cycle in five specified profiles. The WT test was performed on a compacted sample, which was exposed to the moving wheel acting at different loadings. The subsequent rutting depths were logged at various specified cycles, which achieved an in-depth evaluation of the material’s resistance capabilities to rutting [4]. The sample was compacted to 98% MDD and at 80% OMC in 6 layers using a segmented roller compactor. The sample had final dimensions of 500 mm (W) × 700 mm (L) × 300 mm (D).

## 3. Results and Discussion

**Figure 2:**
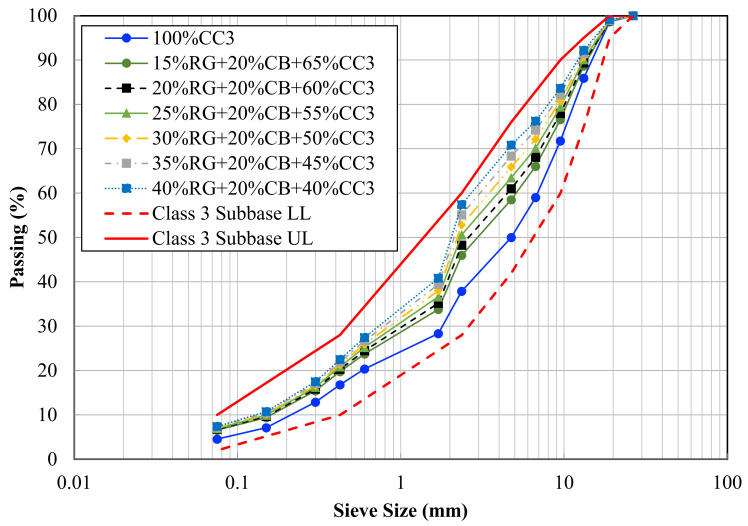
Particle size distribution of the nominated blends.

Figure 2 illustrates the particle size distributions for all the nominated blends along with the lower and upper limits of Class 3, as specified by the state road authority.

The organic content of the 100% CC was less than 1, and for the 100% RG it was 1.2 (Table 1). The average apparent particle density from both fine and coarse aggregates of each material was calculated, and they are reported in Table 1.

The 100% CC had higher particle density compared to the triple blends. A particle is classified as “flaky” when it possesses a least-thickness (dimension) less than 60% of its mean size, as stated in the AS 1141.15 [35]. The material being analyzed for this test was CC. The mass of all “flaky” particles from each sieve were tallied and compared to that of the initial total mass, allowing the overall flakiness index of the selected material to be determined and reported in Table 1. RG water absorption was measured to be less than 1%, while that of the 100% CC was calculated to be in the range of typical quarry materials.

The OMC and MDD of the nominated blends were calculated, and are reported in Table 2. There was a direct relationship between OMC and CC percentage; 100% CC had higher OMC, and 40% RG + 20% CB + 40% CC showed the lowest OMC. The pH values of all the blends are presented in Table 2.

The pH range for the triple blends was 11.23–12.23, which suggests that the parent materials and the triple blends were all alkaline, which is similar to previous studies’ results on the same materials [20]. The LA abrasion test is a commonly specified test for evaluating the resistance of aggregates to abrasion and impact forces. The LA abrasion loss in 100% CC was lower than the maximum LA abrasion value of 40 set by local road authorities for sub-base applications [43].

The permeability results of the nominated triple blends are presented in Table 2. These values describe the nominated blends as a very low-permeability material, based on the hydraulic conductivity classification chart introduced by Terzaghi et al. [44]. When RG was more than 30%, the mixes showed relatively higher permeability due to the high content of non-cohesive fines. The CBR value of 100% CC was high and varied from 229% to 239%. The results of CBR tests for the parent materials are summarized in Table 1 and for all the blends in Table 2. The test results indicate that the CBR value of the triple blends decreased with the addition of RG. All the blends met the local road authority requirement for minimum CBR value of 80% [43].

**Figure 3:**
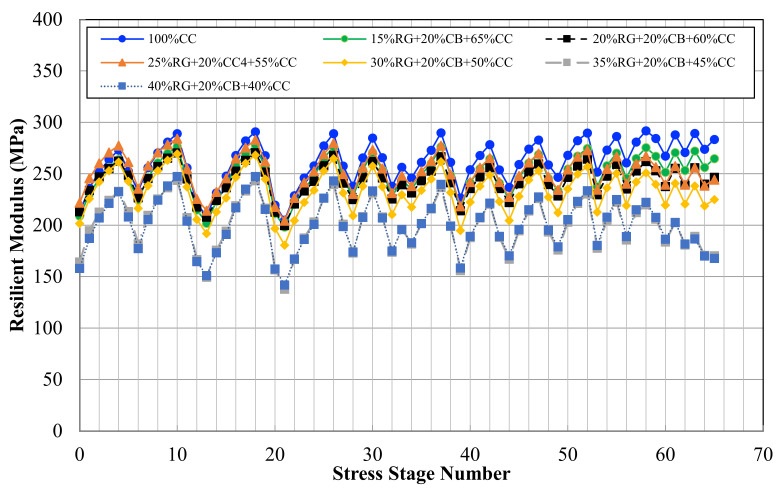
Resilient modulus of the nominated blends.

The results of RLT tests were analyzed to determine the performance of unbound RG/CB/CC triple blends under real traffic loading conditions. Figure 3 shows the results of the resilient modulus test at different stress stages.

**Figure 4:**
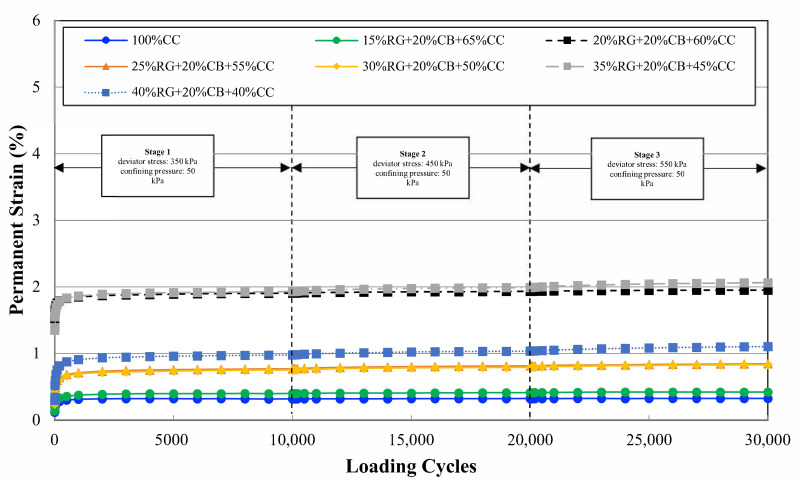
Permanent deformation of the nominated blends.

Based on the results, 100% CC presented the highest resilient modulus range. The permanent deformation test was performed under 50 kPa confining pressure at three different loading stages. Each loading stage comprised 10,000 repeats, which summed to 30,000 cycles for the completion of the permanent deformation test. Figure 4 demonstrates the permanent deformation results for all the blends.

The 15% RG + 20% CB + 65% CC blend demonstrated the lowest permanent deformation after 100% CC. The results of RLT tests are summarized in Table 3 and were in ranges reported in previous studies by Arulrajah et al. [45].

There was a trend of higher permanent deformation with increasing RG content, but a couple of anomalies were noticed within the seven blends. Based on the RLT assessment, 15% RG performed the best among all the blends containing RG. Thus, the 15% RG + 20% CB + 65% CC blend was chosen for the WT test. Austroads [39] specifies a strain limit of 2% under permanent strain loading. All the samples experienced lower values than this limit, except for 35% RG + 20% CB + 45% CC at stage 3 of loading. Among those tested, the blend containing 15% RG + 20% CB + 65% CC had a similar rate of permanent deformation to 100% CC after the first 3000 cycles at each loading stage. Therefore, this blend was chosen for further assessment under the WT loading condition.

**Figure 5:**
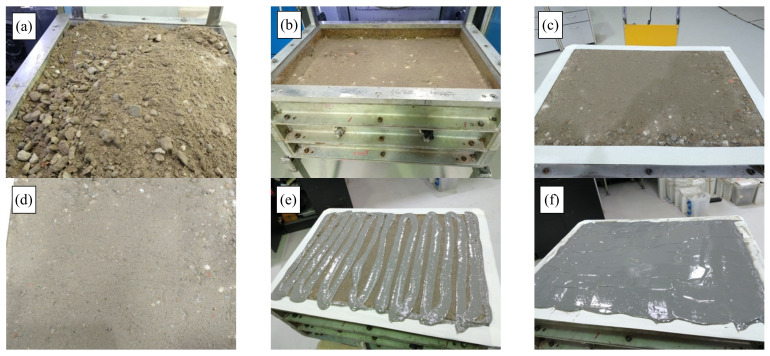
Specimen preparation prior to wheel-tracker (WT) testing (**a**) sample preparation, (**b**,**c**) compaction, (**d**) Specimen surface, (**e**) applying epoxy and (**f**) sealed sample.

A WT test was conducted on the 15% RG + 20% CB + 65% CC sample in a temperature-controlled chamber with a set temperature of 25 °C. The sample size was 500 mm (W) × 700 mm (L) × 300 mm (D). The sample was compacted inside the mold at 80% OMC in six layers using a segmented roller compactor with a maximum load capacity of 30 kN, to reach 98% MDD. Figure 5 shows the different stages of WT sample preparation.

**Figure 6:**
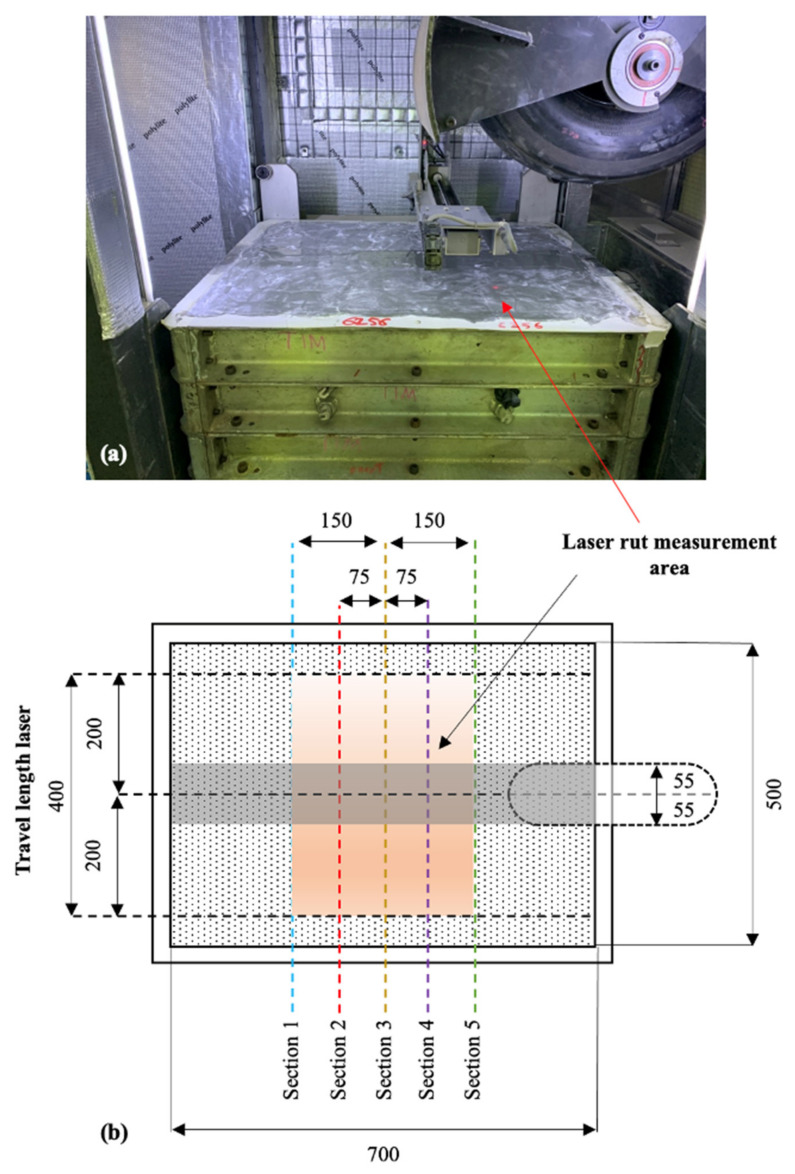
(**a**) Wheel-tracking test setup and (**b**) details and measurement sections, all in mm [4].

The sample was dried back to 70% OMC for the WT test. The sample was sealed with epoxy before the start of the test. The sample was mounted to the temperature control chamber (25 °C) for WT testing. The rutting was recorded using a laser at specific sections (five) during the test to determine the transversal deformation. Figure 6a shows the WT test setup and Figure 6b shows the laser rut sections and measurement area. The WT test was carried out for 40,000 cycles and the mean deformation was recorded after each loading cycle, unless the sample deformed more than 18 mm during the test and failed to meet standards according to Austroads AGP-T054 [42].

**Figure 7:**
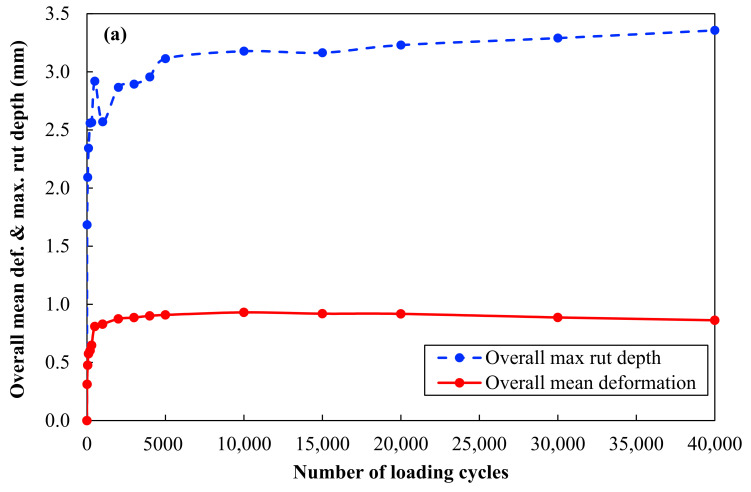

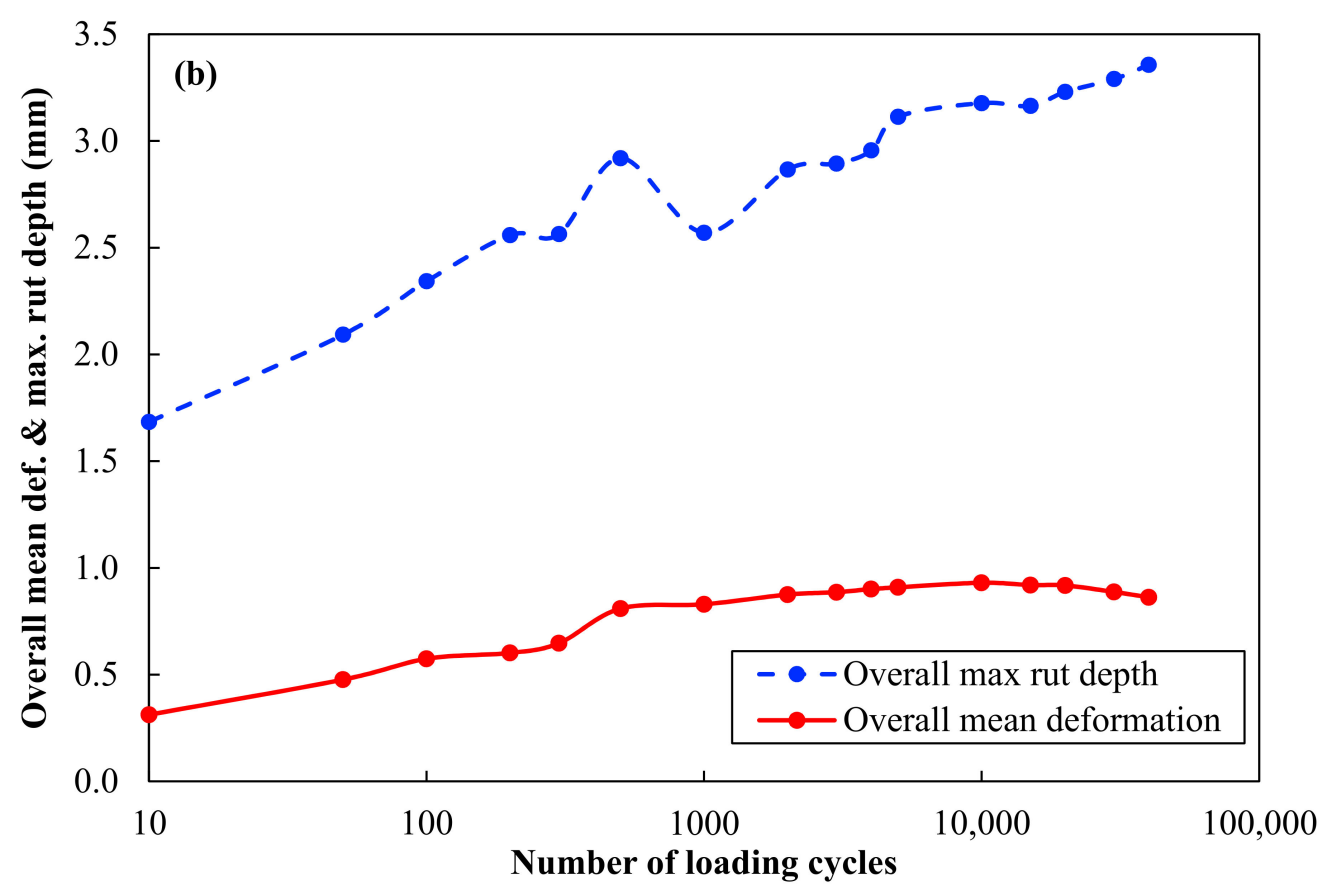
Overall mean surface deformation data and maximum rut depth data for 15% RG + 20% CB + 65% CC: (**a**) Decimal scale and (**b**) semi-logarithmic scale.

The 15% RG + 20% CB + 65% CC sample did not fail after 40,000 cycles of the WT test. The maximum surface deformation and maximum rut depth data are presented in Table 4 and plotted in Figure 7.

For a better understanding, the maximum surface deformation and maximum rut depth in Figure 7a,b are presented against the number of loading cycles in decimal and logarithmic scale, respectively.

**Figure 8:**
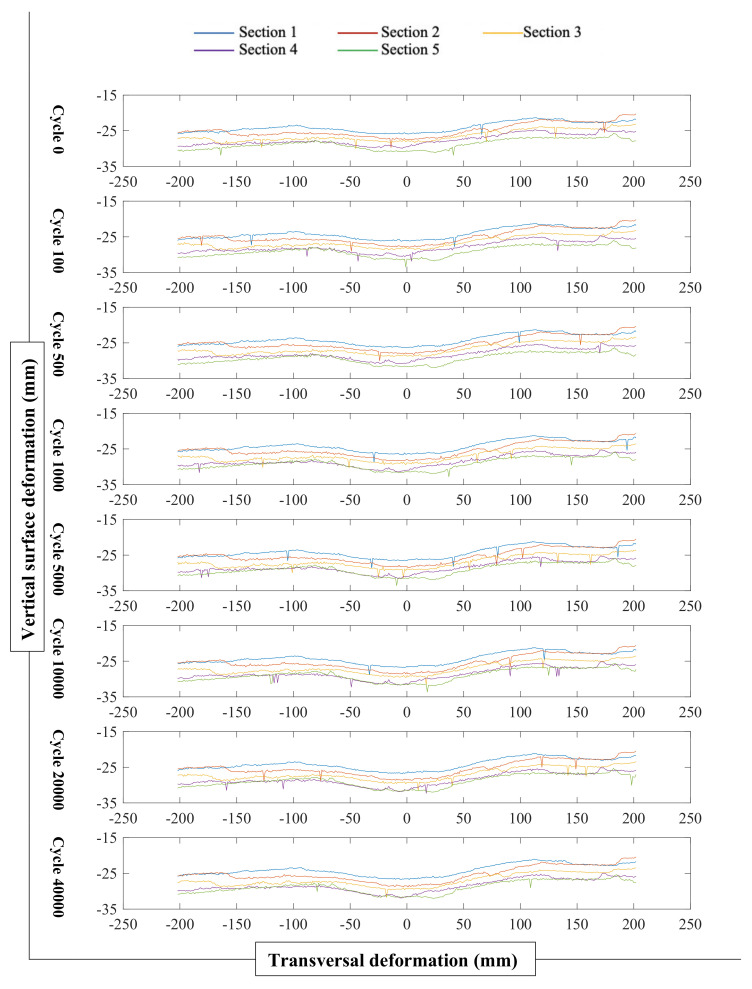
Profile data of all cross sections at different cycles for 15% RG + 20% CB + 65% CC.

Figure 8 presents profile data of all five cross sections at different cycles for 15% RG + 20% CB + 65% CC. As shown in this figure, the vertical surface deformation increased gradually as the number of cycles increased.

**Figure 9:**
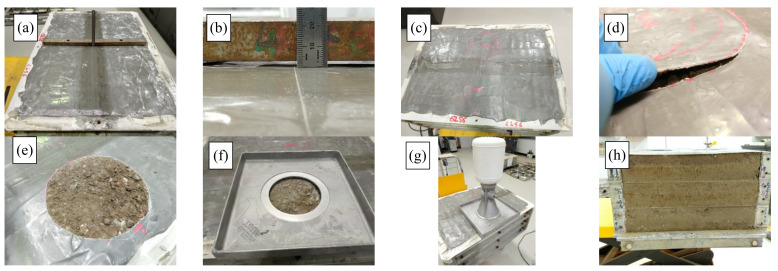
Post WT test rutting and density measurements. (**a**) after test, (**b**) final rut measurement, (**c**) after test specimen, (**d**), (**e**,**f**) sampling after test, (**g**) density check and (**h**) layers after test.

Figure 9 shows the rutting measurement and density check after the WT test. The WT results showed that the 15% RG + 20% CB + 65% CC sample, with maximum surface deformation of 0.9 mm and maximum rut depth of 3.4 mm, performed well after 40,000 cycles and met the Austroads requirements for base/sub-base applications. Austroads [42] specifies a mean deformation of 18 mm as a complete failure of the slab. The mean deformation of sample 15% RG + 20% CB + 65% CC in Table 4 was measured to be far below this threshold.

## 4. Conclusions

The geotechnical characteristics of unbound RG/CB/CC blends consisting of up to 40% RG and 20% CB with CC were evaluated. The particle-size distribution curve of all blends was within the upper and lower limits of Class 3 set by local and state road authority. The CBR values of all blends were well above the minimum requirements specified by the local road authority for blends used in pavement applications.

The results of RLT tests were used to ascertain the performance of unbound RG/CB/CC blends under simulated traffic loading conditions. Based on the RLT assessment, all the nominated blends were found to provide the required resilient modulus value for use as base and sub-base materials. The 15% RG + 20% CB + 65% CC blend demonstrated the lowest permeant deformation after 100% CC under long-term repeated loading. Thus, the 15% RG + 20% CB + 65% CC blend was chosen for the WT test.

The WT results confirmed that the triple blends with 15% RG did not fail during the test. With maximum surface deformation of 0.9 mm and maximum rut depth of 3.4 mm, the sample performed well after 40,000 cycles and comfortably met the Austroads requirement of 18 mm for use in pavement applications.

Based on the engineering properties and permanent deformation results, up to 15% RG can be primarily suggested for incorporation as an accompanying material in unbound roadwork applications. The blends 25% RG + 20% CB + 55% CC and 30% RG + 20% CB + 50% CC exhibited relatively low permanent deformation in the RLT assessment. Thus, subject to the outcomes of future field testing, there may be the potential to increase the percentage of RG added in the blends up to 30% for upcoming projects, as well as to alleviate concerns regarding an increase in the uptake of recycled materials into the marketplace.

## Figures and Tables

**Table 1:** Geotechnical properties of the parent materials.

Engineering Properties		100% CC	100% RG	Typical Quarry ^a^ Materials
Fine content	(%)	4.5	7.8	<10
Sand content	(%)	45.5	92	30–60
Gravel content	(%)	50	0.2	30–60
Apparent particle density	(kN/m^3^)	2.64	2.48	>2
Water absorption	(%)	9.8	<1	6–12
Organic content	(%)	<1	1.2	<5
Flakiness index		19.14	-	<35
LA abrasion loss	(%)	28	-	<40
California Bearing Ratio (CBR)	(%)	239	20	>80
Resilient modulus (M_R_)	(MPa)	205–292	-	125–300

^a^ Data from Maghool, Arulrajah, Horpibulsuk and Du [23].

**Table 2:** Engineering properties of the nominated blends.

Blends	pH	OMC (%)	MDD (kN/m^3^)	CBR (%)	Permeability (m/s)
100% CC	12.4	11.5	1.96	230–248	1.05 × 10^−7^
15% RG + 20% CB + 65% CC	11.87	10.6	1.98	220–257	4.57 × 10^−7^
20% RG + 20% CB + 60% CC	11.78	10.7	1.97	192–228	1.39 × 10^−7^
25% RG + 20% CB + 55% CC	11.64	10.7	1.97	167–182	2.15 × 10^−7^
30% RG + 20% CB + 50% CC	11.51	10.5	1.96	165–202	9.76 × 10^−7^
35% RG + 20% CB + 45% CC	11.37	10.4	1.96	131–157	1.78 × 10^−6^
40% RG + 20% CB + 40% CC	11.23	10.0	1.96	116–147	4.34 × 10^−6^

**Table 3:** Resilient modulus and permanent strain testing for the nominated blends.

Blends	M_R_ (MPa)	Permanent Strain at the End of Each Stage, Microstrain		
		Stage 1	Stage 2	Stage 3
100% CC	205–292	995	1282	1596
15% RG + 20% CB + 35% CC	198–278	1082	1392	1703
20% RG + 20% CB + 40% CC	200–271	1194	1492	1804
25% RG + 20% CB + 35% CC	204–284	1154	1443	1755
30% RG + 20% CB + 30% CC	181–270	1220	1534	1867
35% RG + 20% CB + 25 %CC	138–244	1406	1746	2110
40% RG + 20% CB + 20% CC	142–247	1383	1723	2086

**Table 4:** Overall mean surface deformation and overall maximum rut depth data for 15% RG + 20% CB + 65% CC.

Number of Cycles (N)	Mean Surface Deformation					Mean overall	Maximum Rut Depth					Mean overall
	Cross Section						Cross Section					
	1	2	3	4	5		1	2	3	4	5	
	−150 mm	−75 mm	0 mm	+75 mm	+150 mm		−150 mm	−75 mm	0 mm	+75 mm	+150 mm	
0	0.0	0.0	0.0	0.0	0.0	0.0	0.0	0.0	0.0	0.0	0.0	0.0
10	0.8	0.8	−0.2	0.0	0.2	0.3	2.7	2.9	0.8	1.0	1.0	1.7
50	1.0	1.0	0.0	0.1	0.3	0.5	3.0	3.4	1.4	1.2	1.4	2.1
100	1.1	1.1	0.0	0.3	0.5	0.6	3.3	3.5	1.6	1.6	1.8	2.3
200	1.0	1.1	0.0	0.3	0.5	0.6	3.6	3.8	1.5	2.0	1.9	2.6
300	1.0	1.1	0.1	0.4	0.6	0.6	3.5	3.7	1.7	1.9	2.1	2.6
500	1.1	1.2	0.2	0.7	0.9	0.8	3.6	4.0	2.1	2.4	2.5	2.9
1000	1.1	1.3	0.3	0.8	0.7	0.8	3.5	4.1	1.6	2.3	1.4	2.6
2000	1.1	1.3	0.3	0.9	0.7	0.9	3.5	3.7	2.2	2.6	2.3	2.9
3000	1.1	1.4	0.4	1.0	0.6	0.9	3.6	3.8	2.3	2.6	2.2	2.9
4000	1.1	1.4	0.4	1.0	0.6	0.9	3.7	3.9	2.4	2.7	2.1	3.0
5000	1.2	1.4	0.4	1.0	0.6	0.9	3.8	4.2	2.4	2.8	2.4	3.1
10,000	1.2	1.4	0.5	1.0	0.6	0.9	3.7	4.2	2.7	3.1	2.3	3.2
15,000	1.2	1.4	0.5	1.1	0.5	0.9	3.7	4.1	2.5	3.1	2.4	3.2
20,000	1.2	1.4	0.5	1.1	0.4	0.9	3.9	4.1	3.0	3.0	2.1	3.2
30,000	1.1	1.4	0.5	1.0	0.4	0.9	3.9	4.3	2.8	3.2	2.3	3.3
40,000	1.0	1.4	0.4	1.0	0.4	0.9	3.8	4.5	2.9	3.2	2.4	3.4

## Data Availability

The data presented in this study are available on request from the corresponding author. The data are not publicly available due to Sustainability Victoria regulations.
